# Estigma entre profissionais de saúde da atenção primária: estudo de método misto

**DOI:** 10.1590/0102-311XPT230225

**Published:** 2026-07-20

**Authors:** Aenne Zandonadi Rodrigues Santana, Annelita Almeida Oliveira Reiners, Luciane Almeida Casarin, Rosemeiry Capriata de Souza Azevedo, Joana Darc Chaves Cardoso, Carla Rafaela Teixeira Cunha, Amanda Cristina de Souza Andrade

**Affiliations:** 1 Universidade Federal de Mato Grosso, Cuiabá, Brasil.; 2 Universidade Federal de Rondonópolis, Rondonópolis, Brasil.; 3 Instituto René Rachou, Fundação Oswaldo Cruz, Belo Horizonte, Brasil.

**Keywords:** Estigma Social, Demência, Atenção Primária à Saúde, Profissionais de Saúde, Social Stigma, Dementia, Primary Health Care, Health Care Professionals, Estigma Social, Demencia, Atención Primaria de Salud, Profesionales de la Salud

## Abstract

O objetivo foi analisar como o estigma se apresenta entre profissionais de saúde da atenção primária à saúde, com base nas dimensões do conhecimento, das atitudes e das práticas voltadas às pessoas que vivem com a síndrome demencial. Estudo de métodos mistos, explanatório sequencial, realizado com 122 profissionais de saúde de 91 Unidades de Saúde da Família de Cuiabá, Mato Grosso, Brasil, entre 2023 e 2024. Na etapa quantitativa, foram aplicados os instrumentos *Atenção Sanitária às Demências: A Visão da Atenção Básica* e *Questionário de Atitudes*; na etapa qualitativa, dois grupos focais com 11 participantes foram conduzidos. Na análise dos resultados, os dados quantitativos e qualitativos foram integrados, buscando convergências e divergências interpretativas. Os resultados mostraram insuficiência de conhecimento e de preparo técnico dos participantes sobre a demência, decorrente de ausência de formação específica, sugerindo a presença de estigma estrutural e social. Os profissionais demonstraram concepções equivocadas e estigmatizantes sobre a condição e a pessoa que vive com a demência, e revelaram insegurança profissional e incerteza nas ações de cuidado que contribuem negativamente na realização do diagnóstico oportuno, no tratamento e acompanhamento das pessoas envolvidas. Foi identificada dissonância entre atitudes e práticas, indicando manifestações de estigma implícito. A presença de estigma estrutural, social e implícito entre profissionais da atenção primária à saúde compromete o diagnóstico oportuno, o tratamento e o acompanhamento da pessoa que vive com a demência e familiares, perpetuando práticas centradas na demência e negligência assistencial. Os achados reforçam a necessidade de formação específica e contínua, de educação permanente voltadas à redução do estigma e à promoção do cuidado centrado na pessoa.

## Abstract (en)

This study analyzed how stigma occurs in primary healthcare providers based on dimensions of knowledge, attitudes, and practices targeting people living with dementia syndrome. A mixed-methods, sequential explanatory study was carried out with 122 healthcare providers from 91 Family Health Units in Cuiabá, Mato Grosso State, Brazil, from 2023 to 2024. Its quantitative stage applied the following instruments: *Healthcare for Dementia: A View of Primary Care* and *Attitude Questionnaire*. In total, two focus groups with 11 participants were carried out the qualitative stage of this research. Analysis of the results integrated quantitative and qualitative to find convergences and interpretative divergences. Results showed participants’ insufficient knowledge and technical preparedness regarding dementia due to lack of specific training, suggesting a structural and social stigma. Participants showed stigmatizing misconceptions regarding the condition and people living with it and professional insecurity and uncertainty in care actions, which negatively contribute to patients’ timely diagnosis, treatment, and follow-up. The dissonance between attitudes and practices indicated manifestations of implicit stigma. Structural, social, and implicit stigma in primary healthcare providers compromise timely diagnosis, treatment, and follow-up of patients and family members, perpetuating practices centered on dementia and care neglect. These findings reinforce the need for specific and continuous training to reduce stigma and promote person-centered care.

## Resumen

El objetivo fue analizar cómo se manifiesta el estigma entre los profesionales de atención primaria de salud, con base en las dimensiones del conocimiento, de las actitudes y prácticas dirigidas a las personas que viven con síndrome demencial. Se trata de un estudio de métodos mixtos, explicativo, secuencial, realizado con 122 profesionales de la salud de 91 Unidades de Salud de la Familia en Cuiabá, Mato Grosso, Brasil, entre el 2023 y el 2024. En la etapa cuantitativa, se aplicaron los instrumentos *Atención Sanitaria para las Demencias: La Perspectiva de la Atención Primaria* y el *Cuestionario de Actitudes*; en la etapa cualitativa, se organizaron dos grupos focales con 11 participantes. En el análisis de los resultados, se integraron datos cuantitativos y cualitativos, buscando convergencias y divergencias interpretativas. Los resultados mostraron un conocimiento y una preparación técnica insuficientes entre los participantes con respecto a la demencia, debido a la falta de formación específica, lo que sugiere la presencia de estigma estructural y social. Los profesionales demostraron concepciones erróneas y estigmatizantes sobre la enfermedad y la persona que vive con demencia, y revelaron inseguridad profesional e incertidumbre en las acciones de cuidados que contribuyen negativamente al diagnóstico oportuno, al tratamiento y seguimiento de las personas afectadas. Se identificó una disonancia entre actitudes y prácticas, lo que indica manifestaciones de estigma implícito. La presencia de estigma estructural, social e implícito entre los profesionales de la atención primaria de salud compromete el diagnóstico oportuno, el tratamiento y el seguimiento de la persona que vive con demencia y sus familias, lo que contribuye a perpetuar prácticas centradas en la demencia y a la negligencia asistencial. Los hallazgos refuerzan la necesidad de una formación específica y continua, de una educación permanente destinada a reducir el estigma y a promover el cuidado centrado en la persona.

## Introdução

A demência é uma síndrome neurodegenerativa que compromete progressivamente o funcionamento cognitivo e funcional da pessoa que vive com demência, acompanhada de alterações emocionais e comportamentais que afetam profundamente a vida da pessoa, sua família e os serviços de saúde ^1^ ^,^ ^2^ ^,^ ^3^. Reconhecida como uma das principais causas de dependência entre pessoas idosas, constitui um crescente desafio global: estima-se que o número de pessoas que vivem com demência aumente de 57,4 milhões em 2019 para 152,8 milhões em 2050, com cerca de 71% dos casos concentrando-se em países de baixa e média renda, onde o impacto social e econômico tende a ser ainda mais expressivo ^3^ ^,^ ^4^ ^,^ ^5^. Apesar da magnitude do problema, estima-se que cerca de 80% das pessoas com a condição no Brasil permanecem sem ser diagnosticadas.

Um fator que interfere no reconhecimento e na resposta do sistema de saúde é o estigma relacionado à demência ^6^, reconhecido por organizações internacionais como um problema de saúde pública ^3^ ^,^ ^4^ ^,^ ^7^ ^,^ ^8^ ^,^ ^9^. O estigma é um processo social e cultural que atribui características negativas a indivíduos e grupos, resultando em discriminação, exclusão e redução de expectativas ^10^ ^,^ ^11^. No contexto da demência, manifesta-se de forma explícita e implícita, em níveis pessoal, interpessoal, social e institucional, por meio de estereótipos, atitudes negativas e discriminação ^2^, que afetam decisões clínicas, dificultam o acesso ao diagnóstico e comprometem a qualidade do cuidado e do acompanhamento da pessoa que vive com demência e de sua família/cuidador ^12^.

Pesquisas internacionais têm evidenciado que o estigma relacionado à demência entre profissionais de saúde constitui um fator crítico que compromete o diagnóstico precoce, o acesso ao tratamento e o acompanhamento contínuo, repercutindo negativamente na qualidade de vida e saúde da pessoa que vive com demência e de sua família/cuidador ^4^ ^,^ ^8^ ^,^ ^13^. Entretanto, a maior parte desses estudos está concentrada em países de alta renda ^13^ ^,^ ^14^ ^,^ ^15^ ^,^ ^16^ ^,^ ^17^ ^,^ ^18^ ^,^ ^19^, o que limita a compreensão das especificidades socioculturais e estruturais do fenômeno. Em países de baixa e média renda, embora os relatórios internacionais indiquem elevados níveis de estigma entre profissionais de saúde ^4^ ^,^ ^8^, os estudos permanecem incipientes ^20^ ^,^ ^21^.

No Brasil, um estudo apontou que cerca de um terço dos profissionais de saúde considerou a demência como parte natural do envelhecimento, 8,5% concordaram que pessoas que vivem com demência podem representar perigo para outras pessoas e parcela expressiva afirmou não ser essencial comunicar explicitamente o diagnóstico às pessoas acometidas e seus familiares ^22^. E, há estudo emergente voltado a aprimorar o conhecimento, as atitudes e os comportamentos de agentes comunitários de saúde sobre a demência ^23^.

No contexto da atenção primária à saúde (APS), a presença do estigma é especialmente preocupante, visto que, além de representar a principal porta de entrada do Sistema Único de Saúde (SUS), ocupa posição estratégica para assegurar a integralidade e a longitudinalidade do cuidado. Os profissionais da APS, por sua capilaridade territorial, proximidade e vínculo com a comunidade, desempenham um papel central na detecção precoce, no acompanhamento e no suporte contínuo à pessoa que vive com demência e à sua família/cuidador. Entretanto, o Ministério da Saúde reconhece o estigma como um fator multifatorial e estruturante das barreiras ao diagnóstico, tratamento e acompanhamento adequados no país ^6^, e políticas públicas recentes refletem uma preocupação institucional em enfrentar o subdiagnóstico e o preconceito clínico associados às demências ^24^.

O estigma pode manifestar-se de múltiplas formas, sendo compreendido com base em três dimensões inter-relacionadas: conhecimento, atitudes e comportamentos ^25^. Entre os profissionais de saúde, o conhecimento insuficiente pode gerar negligência e/ou atraso no diagnóstico; as atitudes negativas refletir em crenças e emoções negativas em relação às pessoas que vivem com demência e seus familiares/cuidadores, e o comportamento traduzir-se em práticas discriminatórias e excludentes ^4^ ^,^ ^6^ ^,^ ^26^. Abordar essas dimensões é fundamental para melhorar a detecção precoce e a gestão da qualidade do atendimento. Neste estudo, o objetivo foi analisar como o estigma se apresenta entre profissionais de saúde da APS, com base nas dimensões do conhecimento, das atitudes e das práticas voltadas às pessoas que vivem com a síndrome demencial.

## Método

Estudo de métodos mistos com desenho explanatório sequencial que visou à complementaridade e integração de dados entre as abordagens. Considerando a atribuição de tempo (sequencial), peso (quantitativo), combinação (conectados) e teorização (implícita) para esse tipo de desenho, foi definida a primeira etapa quantitativa (Quan) na qual foi conduzido um estudo quantitativo transversal descritivo; e a segunda etapa qualitativa (Qual) em que foi realizado um estudo de abordagem qualitativa com delineamento exploratório-descritivo ^27^. O estudo foi orientado pelo *Mixed Methods Appraisal Tool* (MMAT; Instrumento de Avaliação de Métodos Mistos) ^28^.

A investigação ocorreu na APS, abrangendo 91 Unidades de Saúde da Família (USF) localizadas na área urbana do Município de Cuiabá, capital do Estado de Mato Grosso, localizado na Região Centro-oeste do Brasil. As unidades são compostas por 103 equipes de saúde da família.

A população foi constituída por todos os médicos e enfermeiros atuantes nas USF, totalizando 206 profissionais. Desses, 72 foram excluídos por não atenderem ao critério mínimo de seis meses de atuação na APS e/ou por estarem afastados de suas atividades no período da coleta de dados. Dos 134 profissionais elegíveis, 12 se recusaram a participar da pesquisa. Assim, a amostra final da primeira etapa foi constituída por 122 profissionais, sendo 57 enfermeiros e 65 médicos. A segunda, incluiu 11 profissionais já participantes na etapa quantitativa, selecionados conforme o interesse em participar da etapa qualitativa.

A coleta de dados quantitativa foi realizada entre julho e outubro de 2023. O convite aos profissionais de saúde ocorreu de forma individual nas USF pela pesquisadora principal, após apresentação às chefias das respectivas unidades e com a anuência da Secretaria Municipal de Saúde (SMS). Na ocasião, foram prestadas informações gerais sobre os objetivos e procedimentos do estudo e, mediante manifestação de interesse em participar, os profissionais receberam o Termo de Consentimento Livre e Esclarecido (TCLE), bem como dois instrumentos de coleta de dados autoaplicáveis, em versão impressa: (i) *Atenção Sanitária às Demências: A Visão da Atenção Básica* ^29^ e (ii) o *Questionário de Atitudes* ^30^.

Os dados foram posteriormente digitados em um formulário eletrônico e ajustados para tratamento e análise em um programa estatístico. Foram realizadas análises descritivas de frequência simples (absoluta e relativa). Foi construído um escore de atitude positiva, baseando-se na soma das perguntas q1 a q5 do *Questionário de Atitudes*, em escala Likert de 1 (discordo plenamente) a 5 (concordo plenamente), interpretado da seguinte forma: quanto maior o escore, maior a atitude positiva. De forma análoga, o escore de atitude negativa foi obtido pela soma das perguntas q6 a q10, em que pontuações mais elevadas indicam maior atitude negativa. Para comparar as médias dos escores de atitudes positiva e negativa entre médicos e enfermeiros, foi utilizado o teste t para amostras independentes quando satisfeitas as suposições de normalidade. Quando essas não foram atendidas, utilizou-se o teste não-paramétrico de Mann-Whitney, considerando o nível de 5% de significância.

De forma sequencial, a coleta de dados qualitativa foi realizada em março de 2024, por meio de dois encontros de grupos focais (GF): um com os enfermeiros (duração de 1h30min) e outro com os médicos (1h50min). Considerando o poder da informação, optou-se por não realizar novos, uma vez que o material coletado demonstrou suficiência para responder aos objetivos do estudo. Os GFs foram conduzidos por um grupo de quatro pesquisadoras coordenado pela pesquisadora principal e aconteceram em uma sala da SMS. Cada sessão seguiu uma estrutura composta pelos seguintes momentos-chave: acolhimento dos participantes, apresentação, orientações sobre a dinâmica dos encontros, estabelecimento do *setting*, debate, síntese e encerramento da sessão. O roteiro dos grupos foi elaborado valendo-se dos achados quantitativos, abordando inicialmente questões gerais e, posteriormente, temas específicos emergentes. A gravação dos encontros foi transcrita, organizada por códigos e submetida à análise de conteúdo do tipo temática ^31^.

A integração dos dados ocorreu em dois níveis. No nível metodológico, os achados das etapas quantitativa e qualitativa foram relacionados por meio da conexão entre os resultados (conexão). No nível dos resultados, realizou-se a interpretação integrada, atribuindo significados aos achados previamente analisados e identificando convergências e divergências entre as etapas (interpretação).

Os dados analisados neste artigo integram o mesmo *corpus* empírico da pesquisa intitulada *Conhecimento, Atitudes e Práticas de Profissionais de Saúde Relacionados à Demência: Um Estudo Misto*, aprovada pelo Comitê de Ética em Pesquisa da Universidade Federal de Mato Grosso (UFMT; parecer nº 5.955.667). O presente artigo apresenta interpretação inédita e contribuições teóricas originais.

## Resultados

O estudo quantitativo foi realizado com 122 profissionais de saúde, dos quais 46,72% (n = 57) eram enfermeiros e 53,27% (n = 65) médicos. Entre os enfermeiros, as idades variaram de 28 a 66 anos, com média de idade 42,52 anos (desvio padrão - DP = 7,58). Entre os médicos, as idades variaram de 26 a 59 anos, com média de 35,69 anos (DP = 7,22).

O sexo feminino predominou em ambas as categorias profissionais, correspondendo a 82,45% entre os enfermeiros e 63,08% entre os médicos. O tempo médio de atuação na APS foi 8,28 anos para os enfermeiros e 5,42 anos para os médicos. Em relação à formação na temática, 73,68% dos enfermeiros e 67,7% dos médicos referiram nunca ter participado de capacitação. Ainda assim, a maioria dos profissionais (96,72%) reconheceu a necessidade de formação específica sobre demência (98,25% dos enfermeiros e 95,38% dos médicos).

Nos últimos 12 meses, 52,3% dos médicos relataram ter identificado entre um e quatro casos de demência e 61,4% dos enfermeiros informaram ter atendido durante a consulta de enfermagem entre um e quatro novos casos de demência (Tabela 1).

**Tabela 1:** Processo diagnóstico das demências por médicos e enfermeiros na atenção primária à saúde (APS). Cuiabá, Mato Grosso, Brasil, 2023 (n = 122).

Variáveis	Médicos		Enfermeiros	
	n	%	n	%
Casos de demência detectados no último ano				
Nenhum	7	10,77	-	-
Entre 1 e 4	34	52,30	-	-
Entre 5 e 9	17	26,15	-	-
Entre 10 e 14	6	9,23	-	-
Entre 15 e 20	1	1,53	-	-
Novos casos atendidos em consulta de enfermagem no último ano				
Nenhum	-	-	8	14,03
Entre 1 e 4	-	-	35	61,40
Entre 5 e 9	-	-	6	10,52
Entre 10 e 14	-	-	4	7,01
Entre 15 ou mais			4	7,01
Sinais e sintomas que levam a suspeitar de demência				
Sintomas psicológicos e comportamentais das demências	51	78,46	50	87,72
Comprometimento cognitivo com alterações de memória	59	90,77	49	85,96
Comprometimento cognitivo sem alterações de memória	31	47,69	18	31,57
Comprometimento da capacidade de executar atividades da vida diária	52	80,00	43	75,43
Dificuldades para identificar um caso de demência				
Diferenciar os sinais e sintomas de demência do envelhecimento normal	46	70,76	-	-
Diferenciar os sinais e sintomas de demência da depressão geriátrica	43	66,15	-	-
Pouca utilidade dos exames complementares	23	35,38	-	-
Testes de avaliação cognitiva e funcional utilizados				
*Mini-exame do Estado Mental*	48	73,38	18	31,57
Avaliação cognitiva-funcional global (CDR)	14	21,53	5	8,77
Teste do desenho do relógio	26	40,00	5	8,77
Habitualmente não utilizo nenhum teste para avaliar a função cognitiva	8	12,30	36	63,16
Índice de Katz	14	21,53	3	5,26
Teste de Lawton	9	13,84	4	7,01
Habitualmente não utilizo nenhum teste para avaliar a capacidade funcional	45	69,23	51	89,47
Frequência com que faz o diagnóstico				
Nunca	3	4,62	-	-
Raramente	34	52,30	-	-
Às vezes	24	36,92	-	-
Frequentemente	4	6,15	-	-
Fase da demência no diagnóstico				
Leve	8	12,30	-	-
Moderada	36	55,38	-	-
Grave	6	9,23	-	-
Não diagnóstico, encaminho ao especialista	15	23,08	-	-
Aspectos que dificultam realizar diagnóstico da demência				
Pouca confiança sobre o diagnóstico (dúvidas quanto ao paciente realmente ter uma demência)	32	49,23	-	-
Pensar que o diagnóstico deve ser feito pelo serviço especializado	15	23,07	-	-
Falta de tempo para realizar o processo de diagnóstico (aplicar testes, análises etc.)	49	75,38	-	-
Pacientes encaminhados a especialistas para confirmar o diagnóstico (%)				
< 10	10	15,38	-	-
11-25	4	6,15	-	-
26-50	8	12,31	-	-
51-75	13	20,00	-	-
> 75	29	44,62	-	-

Os sinais e sintomas mais frequentes relacionados à suspeita de diagnóstico de demência mencionados pelos médicos foram o comprometimento cognitivo com alterações de memória (90,77%). Entre os enfermeiros, foram destacados os sintomas psicológicos e comportamentais das demências (87,72%). As principais dificuldades relatadas pelos médicos para identificar casos de demência incluíram a diferenciação entre os sinais e sintomas da doença e do envelhecimento normal (70,76%) e os de depressão geriátrica (66,15%) (Tabela 1).

Em relação aos testes utilizados para identificar casos de demência na prática clínica, o *Mini-exame do Estado Mental* (MEEM) (73,38%) foi o instrumento mais empregado pelos médicos para avaliar a função cognitiva. Entre os enfermeiros, a maioria (63,16%) mencionou não utilizar nenhum teste para essa finalidade. Quanto à avaliação da capacidade funcional, a maioria dos médicos (69,23%) e dos enfermeiros (89,47%) informou não utilizar nenhum teste (Tabela 1).

Com referência à frequência de realização do diagnóstico da demência, 52,3% dos médicos afirmaram raramente estabelecer o diagnóstico, 55,38% referiram diagnosticar com maior frequência os casos na fase moderada e 44,62% declararam encaminhar mais de 75% dos pacientes para os serviços especializados a fim de confirmar o diagnóstico (Tabela 1).

Os principais fatores que dificultam o diagnóstico entre os médicos foram a escassez de tempo para conduzir o processo diagnóstico (75,38%) e a dúvida quanto ao paciente realmente ter uma demência (49,23%) (Tabela 1).

A maioria dos médicos (47,7%) relatou raramente informar o diagnóstico ao paciente e 41,54% afirmaram raramente comunicá-lo aos familiares. O principal motivo apontado para a não divulgação do diagnóstico foi a percepção de que o paciente não teria capacidade suficiente para compreender a informação (50,77%).

Quanto ao acompanhamento das pessoas que vivem com demência, 70,77% dos médicos e 73,69% dos enfermeiros responderam que realizam entre uma e quatro consultas mensais a estes pacientes. A principal dificuldade referida pelos médicos para o tratamento e seguimento na APS foi a prescrição e o manejo dos fármacos específicos (61,54%) (Tabela 2).

**Tabela 2:** Acompanhamento e tratamento da pessoa que vive com a demência e cuidador por enfermeiros e médicos da atenção primária à saúde (APS). Cuiabá, Mato Grosso, Brasil, 2023 (n = 122).

Variáveis	Médicos		Enfermeiros	
	n	%	n	%
Consultas mensais à pessoa que vive com a demência				
Nenhuma	4	6,15	12	21,05
Entre 1 e 4	46	70,77	42	73,69
Entre 5 e 9	11	16,93	0	0,00
Entre 10 ou mais	4	6,15	3	5,26
Dificuldades para o tratamento e acompanhamento da pessoa que vive com a demência				
Tratamento com fármacos específicos (anticolinesterásicos e/ou memantina)	40	61,54	-	-
Necessidades de apoio do cuidador e/ou da família	39	60,00	-	-
Acompanhamento e tratamento da pessoa que vive com a demência requerem uma grande quantidade de tempo, não disponível na APS	34	52,31	-	-
Dificuldades para o tratamento e acompanhamento da pessoa que vive com a demência grave				
Sim	56	86,15	51	89,47
Não	9	13,85	6	10,53
Motivos das dificuldades				
O tratamento farmacológico é complexo	37	56,92	-	-
Exigem muitas visitas domiciliárias	15	23,07	9	15,79
Não há suporte de atendimento especializado	43	66,15	46	80,70
Suas demandas não são solucionáveis na APS	23	35,38	24	42,10
Por serem pacientes difíceis	-	-	13	22,80
Pacientes encaminhados a especialistas para controle de distúrbios comportamentais (%)				
< 10	7	10,77	-	-
11-25	11	16,92	-	-
26-50	14	21,54	-	-
51-75	7	10,77	-	-
> 75	25	38,46	-	-
Programação de visitas domiciliárias				
Sim	39	60,00	38	66,66
Não	26	40,00	19	33,33
Acompanhamento específico para o cuidador da pessoa que vive com a demência				
Nunca	14	21,53	13	22,80
Raramente	31	47,69	14	24,56
Às vezes	11	16,92	24	42,10
Frequentemente	9	13,84	6	10,52

A maioria dos médicos (86,15%) e enfermeiros (89,47%) relatou dificuldades para tratar e acompanhar pessoas com demência em estágio grave. Para ambos os profissionais, a falta de suporte de atendimento especializado foi o principal motivo (66,15% entre médicos e 89,47% entre enfermeiros). Os médicos destacaram a complexidade do tratamento farmacológico (56,92%), já os enfermeiros ressaltaram que muitas demandas dos pacientes não são solucionáveis no âmbito da APS (42,1%) (Tabela 2).

Entre os médicos, 38,46% afirmaram encaminhar mais de 75% dos pacientes para os serviços especializados para controle dos distúrbios comportamentais, sendo o neurologista o profissional mais frequentemente referido como responsável por este atendimento (89,23%) (Tabela 2).

Em relação ao cuidado domiciliar, a maioria dos enfermeiros (66,66%) e médicos (60%) mencionou realizar visitas domiciliares de forma habitual para o tratamento e acompanhamento das pessoas que vivem com demência. No que diz respeito ao acompanhamento do cuidador da pessoa que vive com demência, 47,69% dos médicos e 42,1% dos enfermeiros informaram raramente ou apenas ocasionalmente incluir o cuidador no planejamento do cuidado (Tabela 2).

Entre as funções da equipe de enfermagem no acompanhamento das demências, os enfermeiros atribuíram maior prioridade ao apoio às necessidades do cuidador e/ou da família (89,47%), ao controle da(s) comorbidade(s) apresentada(s) pelo(a) paciente (84,21%) e avaliação periódica do comprometimento funcional (84,21%).

Os resultados da comparação das médias dos escores de atitudes positivas e negativas indicam que o grupo de profissionais de saúde participantes do estudo apresentou média de atitudes positivas maior do que a de atitudes negativas, com menor variação (Tabela 3).

**Tabela 3:** Médias agrupadas das atitudes dos profissionais de saúde frente a pessoas com demência e familiares. Cuiabá, Mato Grosso, Brasil, 2023 (n = 122).

Atitude	Enfermeiros e médicos	Mínimo-máximo
	Média agrupada (DP)	
Positiva	21,42 (± 2,33)	16-25
Negativa	14,01 (± 3,34)	6-21

DP: desvio padrão.

Nota: média mais alta indica mais atitudes positiva e negativa.

Em relação às afirmativas de 1 a 5 do questionário, que abordam atitudes positivas, 63,12% dos profissionais concordam plenamente que “muito pode ser feito para melhorar a qualidade de vida de cuidadores de pessoas que vivem com demência”; 49,18% concordam que “as famílias preferem ser informadas a respeito da demência de seu parente o mais rápido possível”; 64,75% concordam plenamente que “muito pode ser feito para melhorar a qualidade de vida das pessoas que vivem com demência”; 45,08% concordam plenamente que “fornecer diagnóstico geralmente é mais útil do que prejudicial” e 40,16% concordam que “a demência é mais bem diagnosticada em serviços especializados”.

Nas afirmativas de 6 a 10, que abordam atitudes negativas, 33,61% discordam que “as pessoas que vivem com demência podem esgotar recursos com resultado pouco positivo”; 40,98% discordam que “é melhor conversar com o paciente utilizando eufemismos”; 41,8% discordam que “tratar a demência costuma ser mais frustrante do que gratificante”. Mais da metade (58,2%) discorda que “não vale a pena direcionar as famílias para serviços especializados quando elas não querem usá-los”. Por fim, 41,8% discordam que “a equipe da APS tem um papel muito limitado no cuidado de pessoas que vivem com demência”.

Com base nesses resultados, buscou-se aprofundar a compreensão acerca das concepções dos enfermeiros e médicos sobre a demência, as pessoas que vivem com demência e suas famílias/cuidadores, assim como a forma com que essas concepções se relacionam com o conhecimento, as atitudes e as práticas no contexto da APS. Para isso, foi conduzida a etapa qualitativa, com 11 profissionais de saúde da APS (cinco enfermeiros e seis médicos). A análise das falas possibilitou a identificação de três categorias temáticas principais: (1) “Concepções dos profissionais de saúde sobre a demência, a pessoa que vive com demência e a família”, abrangendo as concepções relacionadas à demência, à pessoa que vive com demência e à família/cuidador e sentimentos dos profissionais de saúde em relação à demência e à pessoa que vive com demência; (2) “Processo diagnóstico da demência na APS”, que emergiu a partir das dificuldades relacionadas ao processo diagnóstico da demência; e (3) “Tratamento e acompanhamento da pessoa que vive com demência na APS”, que reuniu os temas relacionados às concepções e dificuldades sobre o tratamento e o acompanhamento da demência (Quadro 1).

**Quadro 1:** *Joint display* da conexão dos resultados quantitativos e qualitativos. Cuiabá, Mato Grosso, Brasil, 2025.

DADOS QUANTITATIVOS	DADOS QUALITATIVOS
CONHECIMENTO Análise integrada: Profissionais de saúde possuem conhecimento insuficiente acerca da síndrome demencial e falta de preparo técnico para conduzir o processo diagnóstico e o cuidado continuado de pessoa que vive com a demência	
73,68% dos enfermeiros e 67,7% dos médicos nunca participaram de treinamentos nessa área	“*...a própria formação médica. A gente nunca teve nenhum treinamento. Eu tenho 15 anos que eu estou na Secretaria de Saúde* (...)*. Eu nunca tive nenhuma formação da Secretaria voltada para essa parte*” (PS4) “*...e eu acho que o terceiro pilar é a questão em como conduzir o tratamento. Beleza, você conseguiu, fez o diagnóstico, tem a ressonância ali, e aí você vai ter, não digo capacidade, mas treinamento para poder iniciar um tratamento, para saber que medicação?*” (PS3)
Incerteza na diferenciação diagnóstica entre os sinais e sintomas da demência e o envelhecimento normal (70,76%) e a depressão geriátrica (66,15%)	“*...é um processo natural do ser humano* (...) *não é uma coisa só patológica, né? Pode ser patológico, mas é um processo natural do ser humano, todos vão chegar*” (PS8). “*Eu sempre relaciono demência com essa coisa de fim de vida, de velhice.* (...) *eu não sei o termo certinho se é uma degradação, uma regressão social, física ou psicológica*” (PS2) “*E diferencial também, né? Qual a demência? É diferente, uma demência senil, é Alzheimer, qual a demência?*” (PS6)
35,38% não costumam fazer uso de testes complementares − 73,38% dos médicos utilizam o MEEM e 63,16% dos enfermeiros não utilizam; 69,23% dos médicos e 89,47% dos enfermeiros não utilizam os testes de avaliação da capacidade funcional	“*Não sei usar escala, nunca apliquei* [mas] *é mais uma questão de aprender, de aplicar*” (PS9) “*A gente sabe que tem as escalas de atividade de vida diária, de atividade instrumental de vida diária, a mini avaliação no estado mental* (...) *porque a gente sabe que a gente pode fazer isso, que a gente tem esses instrumentos para nos auxiliar, mas infelizmente não é sempre* [que usa]” (PS10)
ATITUDES Análise integrada: Atitudes positivas em relação ao tratamento e acompanhamento da pessoa que vive com a demência e sua família/cuidador são diferentes do discurso sobre o diagnóstico, tratamento e acompanhamento	
41,8% dos profissionais acreditam que tratar a demência é mais gratificante que frustrante	“*Frustrados! Frustrados! Triste! Renegados! Impotente também!*” (PS2) (sentimento do profissional diante das dificuldades)
33,61% dos profissionais consideram que os pacientes com demência não esgotam recursos com resultado pouco positivo	“*Então, para um paciente com diabetes, se a gente prescreve a insulina e atinge a meta, ótimo. Se para um paciente que tem hipotireoidismo, e aí a gente corrige esse hipotireoidismo, viu o resultado do exame, ótimo. Aquela sensação de caixinhas, que a gente vai fazendo um checklist. Agora, na parte da demência é sempre aquela caixinha aberta que, às vezes, a gente não está resolvendo. Aí acaba que dá aquela impressão, novamente, que a gente não resolveu o motivo*” (PS5) “*...ele é um paciente problema, trabalhoso, que dá trabalho. Então você vai ter que fazer muito esforço que vai ocupar a sua agenda a mais, porque eu vou ter que fazer mais visitas, correr atrás do suporte*” (PS8)
41,8% não concordam que a equipe da APS tem um papel muito limitado no cuidado de pessoas com demência	“*Então, eu acho muito difícil lidar com doenças neurológicas, porque às vezes a progressão é sempre pior. O paciente, às vezes, não apresenta tanta melhora. Às vezes, no máximo vai estabilizar. Às vezes, vai fazer os cuidados, mas a nossa formação acaba sendo muito curativa. E, muitas vezes, a gente não vai* (...)*. Na verdade, na demência, a gente não vai curar. A gente tem que fazer o cuidado. Então, eu tenho muita dificuldade. Muita dificuldade mesmo*” (PS5)
≈ 65% dos profissionais acreditam que é possível implementar estratégias eficazes para melhorar a qualidade de vida das pessoa que vive com a demência e de seus cuidadores	“*Tem tanta coisa boa para se fazer, né? E aí, por isso que a gente se frustra. Porque a gente sabe que, às vezes, no fim vai voltar para você aquele mesmo paciente, com aquele mesmo problema, com aquela mesma solução que você não vai conseguir resolver.* (...)*. Me sinto impotente*” (PS5)
PRÁTICAS Análise integrada: Profissionais de saúde possuem práticas pouco voltadas ao diagnóstico, tratamento e acompanhamento da pessoa que vive com a demência e sua família/cuidador, e as que realizam são permeadas de dificuldades	
52,3% raramente realizam o diagnóstico; 36,92% o fazem ocasionalmente. Diagnóstico da demência frequentemente em estágio moderado (55,38%)	“*É porque eu acho que chega para a gente muitas vezes precoce! Como chega muito precoce, com muitas queixas, muitas coisas válidas, a gente tem que estabelecer que é uma demência*” (PS1)
75,38% alegam falta de tempo	“*Então, assim, eu tenho 14 pacientes lá fora aguardando e se a gente não consegue dar uma direcionada, você vai ficar ali uma hora naquela consulta. Você não tem uma hora para aquela consulta*” (PS2) “*A gente tem as limitações para os exames complementares. É mais uma etapa, mais uma demanda, demora bastante. Depois que você pode pedir a ressonância*” (PS1)
≈ 50% têm pouca confiança no diagnóstico que realizam, pois têm dúvidas quanto ao paciente realmente ter uma demência	“*A minha dificuldade, quando o paciente chega, assim, muito no começo, eu vejo essa dificuldade de falar, é uma demência mesmo!*” (PS1) “*Eu sei falar tudo o que você quiser de tuberculose, de demência eu não me sinto tão segura assim* (...) *a gente fica meio perdida dentro daquele quadro tão amplo, como fazer essa condução? Inclusive o diagnóstico*” (PS4)
40,16% concordam que a demência é mais bem diagnosticada em serviços especializados. 44,62% dos médicos encaminham mais de 75% dos pacientes para serviços especializados para confirmar o diagnóstico	“*Não, não, a médica encaminhou para o especialista: para o neuro.* (...) *até porque ela fala que* [a demência] *não é da alçada dela*” (PS6)
Dificuldades para o tratamento e acompanhamento da pessoa que vive com a demência e sua família: complexidade do tratamento (56,92), indisponibilidade de medicamentos específicos (61,54%), escassez de tempo nas consultas (52,31%), e ausência de apoio multiprofissional e de retaguarda especializada, especialmente em casos de demência grave (66,15% dos médicos e 80,7% dos enfermeiros)	“*Eu acho que no fundo a gente tem muita dificuldade de ter essa equipe multidisciplinar. A gente não consegue fazer essa troca. Então isso faz a gente ficar sozinho ali.* [Também] *não tem remédio na unidade!* (...) *a gente vê uma dificuldade de acesso também ao especialista neurologista*” (PS4)
Mais de 60% dos profissionais afirmam que habitualmente programam visitas domiciliares aos pacientes e suas famílias, mas 47,69% dos médicos 42,1% dos enfermeiros não planejam as visitas frequentemente	“*Às vezes quando tem que fazer visita eu falo assim: ‘Ah, eu tenho que ir lá ver aquele paciente’* (...)*. Então, você já pensa: ‘Ai, aquela visita!!!’ Parece como se fosse coisa de outro mundo. Como se você tem que fazer uma preparação psicológica para isso. Então, acontece!*” (PS3)
60% consideram que as necessidades dos cuidadores é um fator dificultador para o tratamento e acompanhamento das pessoas que vivem com a demência na APS	“*Então, essa questão de sobrecarga de família acaba ficando mesmo uma pessoa responsável e às vezes acaba nos faltando mesmo mecanismos para lidar, para conseguir auxiliar*” (PS2) “*Eu acho que uma questão minha, maior assim, não é com o paciente. Acho que a questão maior, de uma forma geral, quando a gente faz visitas para os pacientes é muitas vezes a família* (...) *sempre coloca empecilhos*” (PS4)

APS: atenção primária à saúde; MEEM: *Mini-exame do Estado Mental*; PS: profissional de saúde.

## Discussão

Assim como em outros estudos ^6^ ^,^ ^26^ ^,^ ^32^ ^,^ ^33^ ^,^ ^34^ ^,^ ^35^, esta investigação evidenciou insuficiência de conhecimento e preparo entre os profissionais de saúde para reconhecer sinais de demência e utilizar os recursos diagnósticos e terapêuticos disponíveis na APS. Isso aparece como um dos dificultadores para o diagnóstico oportuno da síndrome demencial, bem como para o tratamento e o acompanhamento da pessoa que vive com demência e de sua família/cuidador. Esse contexto pode refletir a presença de estigma sobre a demência entre os profissionais. Evidências sugerem que a insuficiência de conhecimento sobre o processo de identificação, diagnóstico e manejo clínico dessa condição favorece a manutenção de crenças estigmatizantes e de atitudes discriminatórias no âmbito assistencial ^4^ ^,^ ^15^ ^,^ ^21^ ^,^ ^24^.

Esse contexto de conhecimento insuficiente está relacionado a fragilidades nos processos formativos e de educação permanente. O domínio teórico e prático acerca das síndromes demenciais advindo da formação acadêmica, indispensável à atuação qualificada dos profissionais de saúde, deveria ser assegurado tanto pelas instituições responsáveis pela formação acadêmica inicial quanto pelas instituições de saúde, por meio da educação permanente. Todavia, observa-se uma lacuna significativa na abordagem da temática demência nos currículos formais e na implementação de estratégias sistemáticas de atualização profissional ^36^ ^,^ ^37^.

Tais lacunas formativas não se restringem à dimensão curricular, mas refletem também aspectos culturais e simbólicos presentes nas próprias instituições de ensino e de saúde. Essa fragilidade institucional pode estar enraizada em formas de estigma social e profissional que perpassam a construção de saberes e as práticas sobre a demência. Valores, crenças e normas socioculturais que vinculam a demência à inutilidade, dependência e incapacidade podem influenciar a formulação de currículos, políticas e programas de formação profissional. Como consequência, observa-se a omissão ou o tratamento superficial de conteúdos teóricos e práticos sobre a demência, o que reforça lacunas na qualificação das equipes. Essa lacuna formativa, por sua vez, pode favorecer a reprodução de estereótipos negativos e de atitudes estigmatizantes direcionadas às pessoas que vivem com demência e seus familiares/cuidadores. Evidências nesse sentido foram reportadas em estudo conduzido na Polônia, no qual baixos níveis de conhecimento sobre demência entre estudantes de medicina estiveram associados a maiores atitudes estigmatizantes ^14^.

Essas representações estigmatizantes, construídas desde a formação, tendem a se perpetuar no exercício profissional, influenciando a forma com que os profissionais de saúde percebem e interagem com a pessoa que vive com demência ^38^. O estigma, nesse contexto, impacta a abordagem diagnóstica e assistencial, podendo resultar em atrasos ou negligência no diagnóstico, práticas discriminatórias e desvalorização do papel das famílias e cuidadores ^6^. Tais atitudes constituem uma barreira significativa à oferta de cuidado integral às pessoas que vivem com essa condição ^15^ ^,^ ^18^ ^,^ ^21^. Esse contexto favorece práticas assistenciais fragmentadas e centradas na doença, em detrimento de uma abordagem integral, fundamentada no cuidado centrado na pessoa e no reconhecimento de seus direitos e potencialidades, como preconizado pelas diretrizes contemporâneas de atenção à saúde das pessoas que vivem com demência e suas famílias ^6^ ^,^ ^9^ ^,^ ^34^.

Além disso, o estigma internalizado pelos profissionais repercute sobre a organização dos serviços de saúde, comprometendo a estruturação dos cuidados destinados às pessoas que vivem com demência ^38^ ^,^ ^39^ ^,^ ^40^ ^,^ ^41^. Essa influência contribui para a invisibilidade das pessoas que vivem com demência nos serviços, bem como para a ausência de fluxos adequados de triagem, encaminhamento e acompanhamento ^8^ ^,^ ^20^ ^,^ ^25^. Como consequência, os profissionais de saúde permanecem sem suporte técnico e institucional adequado para lidar, de forma qualificada e ética, com a síndrome e com os sujeitos envolvidos no processo de cuidado ^20^ ^,^ ^42^ ^,^ ^43^.

Tal contexto contribui para a invisibilidade da condição no âmbito educacional e assistencial, configurando-se como expressão de estigma estrutural no campo da saúde ^8^, que se manifesta por meio de práticas institucionais e rotinas clínicas que marginalizam e silenciam as necessidades da pessoa que vive com demência e de sua família/cuidador ^25^. De forma cíclica, o estigma estrutural também reforça o estigma do profissional de saúde, ao não lhe fornecer a base teórica e prática necessária para atuar frente à síndrome demencial e às pessoas envolvidas no cuidado.

Adicionalmente, a consolidação desses estigmas pode ser influenciada pela forma como as políticas públicas específicas são concebidas, bem como pela maneira na qual o sistema de saúde se estrutura para o cuidado direcionado à síndrome demencial, à pessoa que vive com demência e sua família/cuidador ^20^ ^,^ ^23^ ^,^ ^35^ ^,^ ^44^. Com o intuito de enfrentar a insuficiente compreensão das demências e o subdiagnóstico associado ao estigma, o Ministério da Saúde publicou um relatório nacional ^6^ e um fluxograma de apoio à detecção precoce na APS ^24^. Contudo, a efetiva redução do estigma depende da implementação concreta dessas políticas e da organização do sistema de saúde em consonância com esse propósito.

Neste estudo, a maioria dos profissionais de saúde apresentou atitudes positivas frente à pessoa que vive com demência e seus familiares, resultado consistente com o de outras pesquisas realizadas com profissionais de saúde ^45^ ^,^ ^46^ e diferente de outros estudos ^26^ ^,^ ^32^. Todavia, apesar da predominância de atitudes positivas, evidenciada pela média superior em relação às atitudes negativas, a análise integrada dos dados revelou discrepâncias entre as atitudes assinaladas e as práticas relatadas pelos profissionais de saúde no cuidado à pessoa que vive com demência e seus familiares/cuidadores, indicando que a empatia e a predisposição favoráveis nem sempre se traduzem em práticas assistenciais consistentes.

Essa divergência entre atitudes e práticas sugere que, apesar de uma apresentação atitudinal positiva, elementos subjacentes podem influenciar o comportamento profissional, resultando em ações que não refletem plenamente as disposições atitudinais expressas. Isso pode ser compreendido como resultante de viés implícito, ou seja, pensamentos, sentimentos, associações não conscientes que podem influenciar, sem que se perceba ou tenha intenção, os julgamentos, atitudes e comportamentos sobre pessoas ou grupos sociais em contextos sociais e profissionais ^47^. Estudos apontam que o viés implícito entre profissionais de saúde pode afetar negativamente o relacionamento profissional/paciente, a disposição para o cuidado centrado na pessoa, o diagnóstico precoce e a adesão a diretrizes baseadas em evidências ^47^ ^,^ ^48^.

De outro modo, essa incongruência entre atitude e prática pode também ser interpretada como uma manifestação do estigma, em que as atitudes dos profissionais expressam um conflito entre crenças e práticas, revelando um discurso orientado por um ideal normativo. Nessa lógica, os profissionais reproduzem verbalmente aquilo que social e culturalmente acreditam ser esperado, ajustando seu discurso a padrões socialmente prescritos, mas sem que isto represente uma transformação efetiva de práticas ^10^. O que se observa, portanto, é que nem sempre as ações praticadas refletem o discurso sobre a relevância do cuidado humanizado e da participação da família no tratamento.

No contexto da APS, tal fenômeno torna-se particularmente relevante, em que o estigma pode se manifestar por meio da reprodução de preconceitos e estereótipos associados à demência que, mesmo que de forma inconsciente, podem influenciar a percepção da condição e comprometer o comportamento profissional e a qualidade da interação clínica. Evidências apontam que muitos profissionais, assim como os deste estudo, tendem a conceber a demência como parte natural do envelhecimento, uma condição irreversível e sem possibilidades terapêuticas significativas ^8^ ^,^ ^49^. Tais representações estigmatizantes obscurecem a compreensão da demência como condição patológica diferenciada e passível de intervenção, reforçando a ideia de que tais pacientes são menos passíveis de receber atenção especializada ^36^. Consequentemente, essas crenças e atitudes podem atrasar o diagnóstico, desestimular a comunicação franca e empática com pacientes/famílias e reduzir sua participação nas decisões e dificultar o engajamento terapêutico ^19^ ^,^ ^22^.

Nesta pesquisa, a insuficiência de conhecimento dos profissionais de saúde e o indicativo da presença de estigma atuam como um importante fator de insegurança nas práticas desses trabalhadores. Na condução do tratamento e nos cuidados contínuos à pessoa que vive com demência e à sua família/cuidador, eles se veem com dificuldades e desafios na aplicação das diretrizes do tratamento e acompanhamento da síndrome demencial, o que resulta em incerteza na seleção das intervenções mais adequadas. Essa incerteza diagnóstica leva os profissionais a subestimarem a relevância do reconhecimento precoce da demência, perpetuando a negligência e comprometendo o acesso da pessoa que vive com demência e de sua família/cuidador às orientações e ao planejamento adequado dos cuidados futuros ^50^.

No Brasil, é limitado o acesso ao diagnóstico, tratamento e apoio formal às pessoas que vivem com demência e às suas famílias/cuidadores ^40^. Associado à escassez de recursos terapêuticos, ao déficit de apoio institucional e à sobrecarga de demandas enfrentadas pelos profissionais na APS, esse cenário pode favorecer atitudes de evitação e distanciamento em relação à pessoa que vive com demência e seus cuidadores ^19^ resultando, como evidenciado neste estudo, em acompanhamento insuficiente e lacunas no cuidado que comprometem a qualidade da assistência e perpetuam práticas discriminatórias, como a redução de visitas domiciliares, a ausência de monitoramento contínuo da evolução clínica e a escassez de orientações direcionadas à família.

### Forças e limitações

Até onde se tem conhecimento, este é o primeiro estudo a empregar uma abordagem de métodos mistos para analisar a presença do estigma relacionado à síndrome demencial entre profissionais da APS, abrangendo as dimensões do conhecimento, das atitudes e das práticas em relação às pessoas que vivem com demência e seus familiares/cuidadores. Ao integrar dados quantitativos e qualitativos, a investigação alcança maior profundidade analítica e amplia a compreensão do estigma em seus níveis individual, relacional e institucional, conferindo originalidade e relevância científica aos achados.

Apesar das contribuições, algumas limitações devem ser reconhecidas. O uso do instrumento *Questionário Atenção Sanitária às Demências: A Visão da Atenção Básica*, cujas questões sobre atitudes não apresentavam delimitação conceitual clara, o que pode ter afetado a precisão dos achados. Essa limitação foi atenuada pela aplicação complementar do *Questionário de Atitudes*, instrumento validado para a avaliação específica das atitudes. Outra limitação diz respeito a não inclusão de profissionais das unidades rurais da APS, o que restringe a abrangência contextual dos resultados. Ainda assim, o estudo mantém relevância científica e prática, ao abordar o cenário urbano, onde se concentram a maior parte da população atendida e as demandas crescentes relacionadas à demência. Investigações futuras poderão ser direcionadas à análise do estigma entre profissionais de saúde no contexto rural de atendimento às pessoas que vivem com demência.

## Conclusão

Os resultados deste estudo demonstram a insuficiência de conhecimento teórico e prático dos profissionais de saúde da APS sobre a síndrome demencial, decorrente da ausência de formação específica e continuada, que contribui para a presença de concepções estigmatizantes, insegurança e incerteza no comportamento direcionado às pessoas que vivem com demência e seus família/cuidadores e para práticas centradas na doença, em detrimento do cuidado centrado na pessoa. Os achados também evidenciam uma dissonância entre as atitudes assinaladas e as práticas declaradas dos profissionais de saúde, indicando que nem sempre as ações praticadas refletem o discurso declarado. Associada à escassez de recursos, à falta de apoio institucional e à sobrecarga de trabalho, essa situação favorece o distanciamento profissional e resulta em lacunas assistenciais e práticas discriminatórias. O estigma, de caráter implícito, social e estrutural, repercute negativamente em todas as etapas do cuidado, resultando em atrasos diagnósticos, práticas discriminatórias e desvalorização do papel da família.

A compreensão dos achados deste estudo à luz das diretrizes do SUS evidencia que a insuficiência de conhecimento e a presença de estigma entre os profissionais da APS não se restringem a dimensões individuais, mas refletem lacunas institucionais e sistêmicas na formação e no apoio contínuo às equipes. Nesse sentido, além de melhorar o investimento em conhecimento, destaca-se a importância da Educação Permanente em Saúde como estratégica para promover processos reflexivos e críticos no cotidiano dos serviços, que ressignifiquem a síndrome demencial, a pessoa que vive com demência, sua família/cuidador e as práticas de cuidado, e favoreçam a reorganização dos serviços para o enfrentamento do estigma e promoção de práticas humanizadas e integradas.

A pesquisa fornece subsídios relevantes no sentido de evidenciar as práticas discriminatórias realizadas pelos profissionais de saúde e reforça a necessidade de repensar a organização dos serviços de saúde, sobretudo da APS, no enfrentamento ao estigma institucional. Novos estudos longitudinais e de intervenção poderão ampliar a compreensão sobre estratégias efetivas de redução do estigma e aprimoramento da atenção às pessoas com demência na APS.

## Data Availability

Os dados de pesquisa estão disponíveis mediante solicitação à autora de correspondência.
